# Supplementary material for: The chemokines CCL22 and CCL17 are a defining feature of type 2 stimulated human lung macrophages and exhibit different metabolic dependencies
Source: Front Immunol. 2025 Oct 13;16:1654717. doi: 10.3389/fimmu.2025.1654717 (PMC12554769; doi:10.3389/fimmu.2025.1654717)
Supplement: Supplementary file 1 [file DataSheet1.pdf]

Supplemental material

Figures

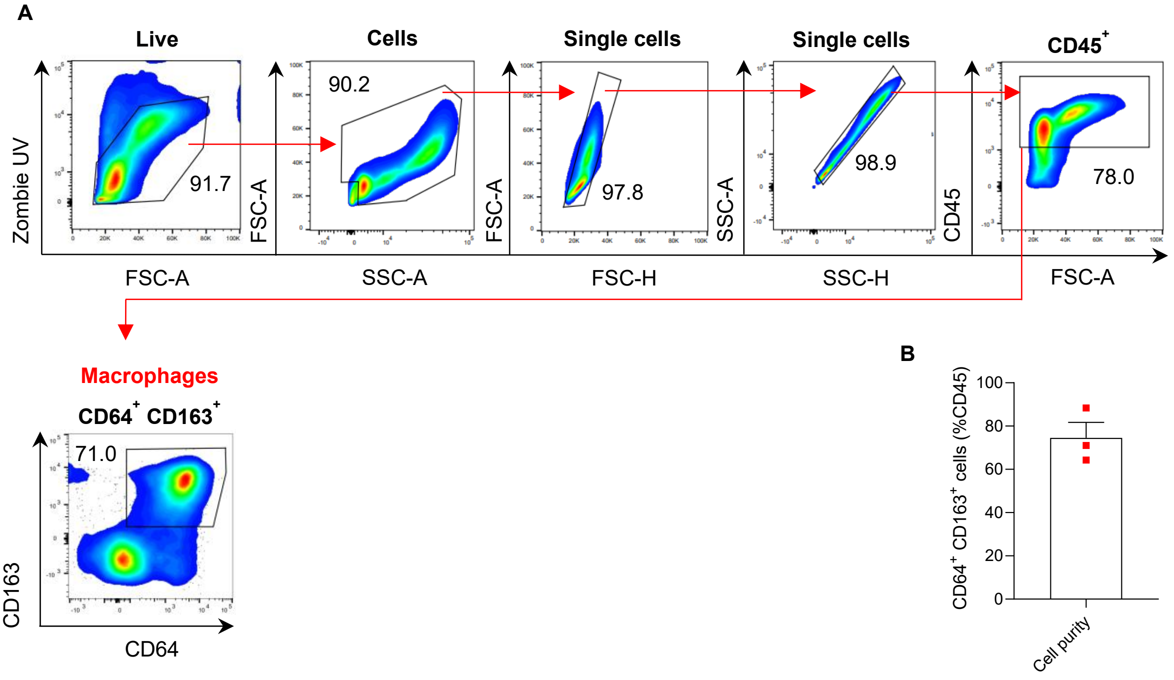

Supplemental Figure 1. Cell purity assessment by flow cytometry.
Schematic illustration of the flow cytometric gating strategy used to assess purity of the macrophages isolated from resected human lung tissue. (A) Cells were gated to identify live (Zombie UV) cells that were single CD45^{+} leukocytes before macrophages were identified as CD64^{+} CD163^{+}. (B) Cell purity of CD64^{+} CD163^{+} human lung macrophages was assessed by flow cytometry before being quantified and expressed as a percentage of CD45.

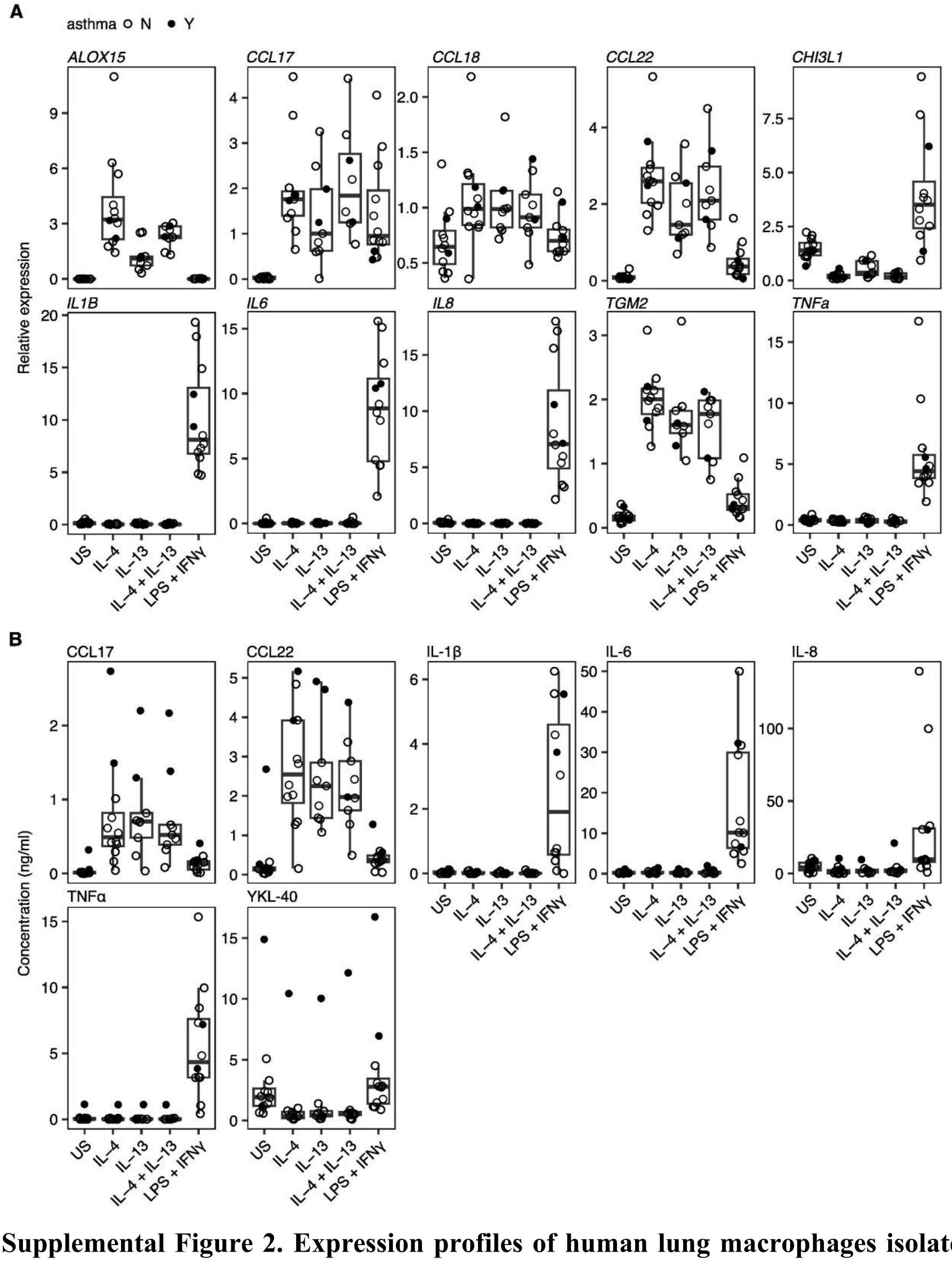

Supplemental Figure 2. Expression profiles of human lung macrophages isolated from non-asthmatic versus asthmatic donors.

(A) Scatter plots showing quantification of mRNA expression in human lung macrophages following stimulation with IL-4, IL-13, IL-4 + IL-13, or LPS + IFNγ. Relative gene expression was normalized to *HPRT* using the $2^{-\Delta CT}$ method. US indicates unstimulated controls. Human lung macrophage responses from non-asthmatics (open circles) and asthmatic (black filled circles) donors. Box plots indicate the median and IQ range, whiskers indicate 1.5x the quartile limit. (B) Scatter plots showing quantification of protein secretion into the supernatant of macrophages stimulated with IL-4, IL-13, IL-4 + IL-13, or LPS + IFNγ, measured by ELISA. Boxes indicate the median and IQ range, whiskers indicate 1.5x the quartile limit. Statistical values were calculated using Wilcoxon two-sample tests and adjusted for multiple testing using

the Holm method. * indicates adjust $P < 0.05$, ** indicates adjust $P < 0.01$, *** indicates adjust $P < 0.001$, **** indicates adjust $P < 0.0001$.

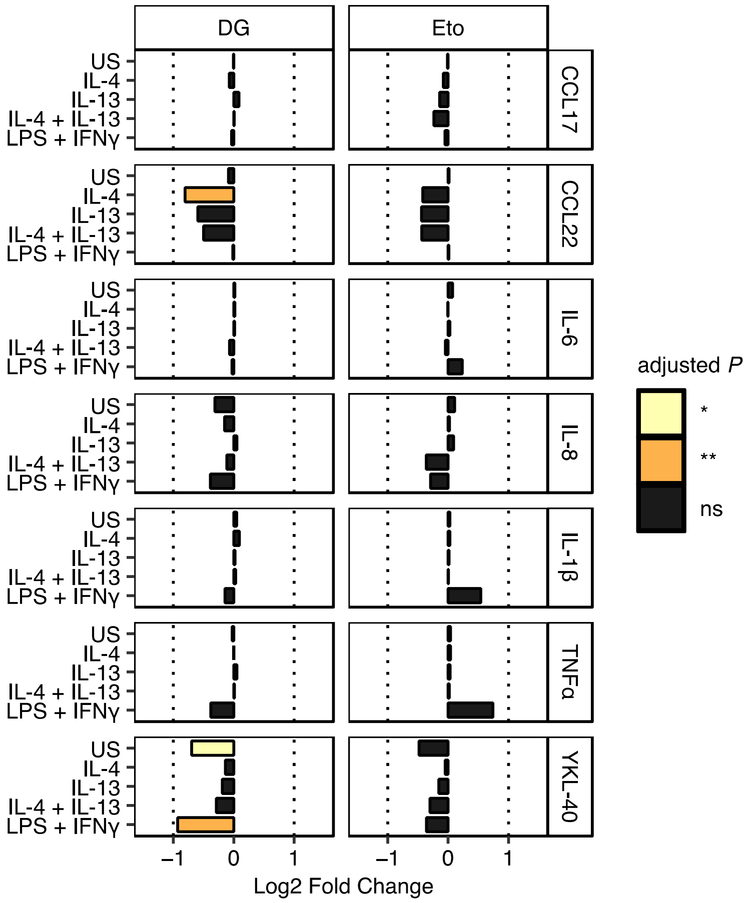

Supplemental Figure 3. Macrophage protein production in the presence of metabolic inhibitors.

Macrophages isolated from resected human lung tissue were stimulated with IL-4, IL-13, IL-4 + IL-13, or LPS + IFNγ and cultured in the presence of metabolic inhibitors, 2-Deoxy-D-glucose (2-DG) or Etomoxir (Eto). The culture supernatant was collected, and the presence of secreted proteins was quantified by ELISA. Bar chart representing Log2 Fold Change in protein concentration for each protein and stimulation condition. Fold change is expressed relative to the matched culture condition without metabolic inhibitor. Bar colour indicates the level of statistical significance. * indicates adjust $P < 0.05$, ** indicates adjust $P < 0.01$.

Supplementary Table 1. ELISA reagents.

Specificity	Conjugation	Clone	Usage concentration	Manufacturer
CCL17 (TARC)	Purified	Poly5230	0.5 µg/ml	Biolegend
CCL22 (MDC)	Purified	-	2 µg/ml	R&D DuoSet
IL-6	Purified	MQ2-13A5	2.5 µg/ml	Biolegend
IL-8	Purified	BH0814	2.5 µg/ml	Biolegend
TNFα	Purified	Mab1	1 µg/ml	Biolegend
YKL-40	Purified	-	1 µg/ml	R&D DuoSet
IL-1β	Purified	-	4 µg/ml	R&D DuoSet
CCL17 (TARC)	Recombinant		1 ng/ml	Biolegend
CCL22 (MDC)	Recombinant		1 ng/ml	R&D DuoSet
IL-6	Recombinant		10 ng/ml	Biolegend
IL-8	Recombinant		100 ng/ml	Biolegend
TNFα	Recombinant		100 ng/ml	Biolegend
YKL-40	Recombinant		2 ng/ml	R&D DuoSet
IL-1β	Recombinant		0.25 ng/ml	R&D DuoSet
CCL17 (TARC)	Biotin	Poly5230	0.5 µg/ml	Biolegend
CCL22 (MDC)	Biotin	-	0.05 µg/ml	R&D DuoSet
IL-6	Biotin	MQ2-39C3	2.5 µg/ml	Biolegend
IL-8	Biotin	BH0840	2.5 µg/ml	Biolegend
TNFα	Biotin	MAb11	0.5 µg/ml	Biolegend
YKL-40	Biotin	-	0.1 µg/ml	R&D DuoSet
IL-1β	Biotin	-	0.15 µg/ml	R&D DuoSet

Supplementary Table 2. Primers.

Gene	Forward (Fwd) or Reverse (Rev)	Sequence	Accession number	Product length
Ccl17	Fwd	GAGCCATTCCCCTTAGAAAGC	NM_002987.3	133
	Rev	GCATTCTTCACTCTCTTGTTGTT		
Ccl22	Fwd	ATCGCCTACAGACTGCACTC	NM_002990.5	620
	Rev	GACGGTTAACGGACGTAATC		
Alox15	Fwd	GGAGCCTTCCTAACCTACAGC	NM_001140.5	143
	Rev	CTCACGATTCCTTCCACATACC		
Tgm2	Fwd	TGTGGCACCAAGTACCTGCTCA	XM_054323960.1	121
	Rev	GCACCTTGATGAGGTTGGACTC		
Ccl18	Fwd	CCCTCCTTGTCCTCGTCTG	NM_002988.4	240
	Rev	GCTTCAGGTCGCTGATGTATT		
Il-6	Fwd	GACAGCCACTCACCTCTTCA	NM_000600.5	120
	Rev	CCTCTTTGCTGCTTTCACAC		
Il-8	Fwd	GCTCTGTGTGAAGGTGCAGT	NM_000584.4	184
	Rev	CCAGACAGAGCTCTCTTCCA		
Tnf-α	Fwd	AGAACTCACTGGGGCCTACA	NM_000594.4	177
	Rev	GCTCCGTGTCTCAAGGAAGT		
Chi3l1	Fwd	GTGAAGGCGTCTCAAACAGG	XM_054333988.1	141
	Rev	GAAGCGGTCAAGGGCATCT		
Il-1β	Fwd	AAAGCTTGGTGATGTCTGG	NM_000576.3	89
	Rev	GGACATGGAGAACACCACT		
Hprt	Fwd	CCTGGCGTCGTGATTAGTGAT	NM_000194.3	131
	Rev	AGACGTTCAGTCCTGTCCATAA		
